# Comparative study of meningeal enhancement in canine and feline otitis media-interna: 3D-gradient-echo vs. fat-suppressed turbo-spin-echo-T1-weighted sequences in MRI

**DOI:** 10.3389/fvets.2025.1664006

**Published:** 2025-09-30

**Authors:** Florian Graf, Matthias Dennler, Katrin Beckmann, Nico Mauri, Mariano Makara, Adriano Wang-Leandro

**Affiliations:** ^1^Clinic for Diagnostic Imaging, Department of Diagnostics and Clinical Services, Vetsuisse Faculty, University of Zurich, Zurich, Switzerland; ^2^Vetimage Diagnostik AG, Oberentfelden, Switzerland; ^3^Neurology Service, Department of Small Animal, Vetsuisse Faculty, University of Zurich, Zurich, Switzerland; ^4^Department of Diagnostic Imaging, WAVES Western Australian Veterinary Emergency & Specialty, Success, WA, Australia; ^5^Department of Small Animal Medicine and Surgery, University of Veterinary Medicine Hannover Foundation, Hannover, Germany

**Keywords:** dixon, fat-suppression, meningitis, otogenic intracranial infection, post-contrast sequence, SPIR

## Abstract

MRI plays an important role in diagnosing meningeal disease in dogs and cats. It remains unclear which T1 sequence most reliably detects meningeal contrast enhancement. The aim of this retrospective study was to compare detection rate, inter- and intra-observer agreement of meningeal enhancement in otitis media-interna between 3D-GRE-T1W and transverse FS-TSE-T1W sequences. MRI studies of dogs and cats with otitis media and interna from 2017 to 2023 including both sequences of interest were collected and supplemented with a control group. Five observers assessed presence or absence of meningeal enhancement twice independently in each sequence and blinded to the diagnosis. Detection rate, inter- and intra-observer agreement were evaluated using Student's T-Test and Fleiss' Kappa test, respectively. Cases from 46 dogs and 74 cats and 21 control cases met the inclusion criteria. On average, the observers detected meningeal enhancement significantly (p < 0.01) more often in the FS-TSE-T1W than in the 3D-GRE-T1W sequence. Significance applied only to cats, but not to dogs. They reached a substantial inter-observer agreement in both sequences (kappa values of 0.701 and 0.735) and a substantial intra-observer agreement independent of the sequence. These results show that highlighting diseased meninges with fat saturation contributes more than a reduction of volume-average artifacts by increasing resolution in the detection of meningeal enhancement in cats and dogs with otitis media and interna. However, over-interpretation of normal meningeal appearance is possible.

## 1 Introduction

Otitis media is frequently observed in dogs and cats, with various underlying causes. Ascending intracranial infections are rare but represent a severe complication that leads to increased morbidity and mortality (1). Accurate early diagnosis is therefore important in the management of these cases. It currently remains unclear which magnetic resonance imaging (MRI) sequence works best for the detection of meningeal enhancement (2, 3).

Otitis media and interna (OMI) is the most common cause of peripheral vestibular syndrome in cats and the second most common cause in dogs (4, 5). In cats otitis media-interna is most commonly associated with inflammation extending through the Eustachian tube (6, 7), while in dogs, otitis media usually originates from otitis externa (8). Central nervous system (CNS) complications of otitis media-interna are infrequent but recognized as a serious problem in animals (9–11). Infections from the middle and inner ear can spread to the brain through various pathways, including erosion of the medial part of the petrous temporal bone, bacterial migration along blood vessels or cranial nerves, and hematogenous dissemination. This can lead to conditions such as neuritis, meningoencephalitis, and/or formation of intracranial abscesses and intracranial empyema (12, 13). Surgical and pathological findings from a previous study suggest that the most likely anatomical route for the entry of organisms from the inner ear leads through the internal acoustic meatus along the vestibulocochlear nerve and blood vessels (9).

The diagnosis of ascending intracranial infections can be challenging, as cerebrospinal fluid (CSF) analysis alone does not always show significant association with meningeal enhancement (6, 14, 15). Therefore, a presumptive diagnosis of intracranial extension is often based on a combination of clinical signs, MRI and CSF findings. MRI represents a crucial, non-invasive method for diagnosing intracranial diseases in small animals (16). Meningeal enhancement in MRI is typically associated with inflammatory or neoplastic infiltration and is considered a key imaging feature in detection of intracranial extension of otitis media-interna (17, 18). Enhancement is deemed pathological if it appears on multiple adjacent slices, shows nodular changes, or if the signal intensity or thickness (>2 mm) is increased. Additionally, serpentine enhancement along the sulci or linear enhancement along the brainstem surface is also considered pathological (2, 19).

One way to improve the detection of meningeal enhancement in MRI involves the use of fat-suppression in gadolinium-enhanced T1-weighted spin echo sequences (2). Fat-suppression can be obtained with short tau inversion recovery (STIR), opposed phase fat-suppression and chemical fat saturation, being the latter the most commonly used.

The Dixon method is based on chemical shift imaging to achieve consistent fat-suppression. It offers several advantages, including improved uniformity in fat-suppression, reduced susceptibility to artifacts, and compatibility with various imaging sequences (such as spin echo and gradient echo) and different weightings (T1, T2 and proton density) (20).

A study from 2011 found a significantly increased chance to obtain a definitive diagnosis of meningeal enhancement with the use of post-contrast chemical fat-suppression in comparison to post-contrast transverse T1-weighted FLAIR sequences (2).

A second way comprises the use of volumetric T1-weighted imaging. During volumetric imaging acquisition, the entire volume of tissue is excited at once and there are no gaps between slices. This offers several benefits compared to traditional 2D imaging, including acquisition of smaller voxels and multiplanar reconstruction. Smaller voxels and the absence of interslice gaps help to reduce partial volume effects and to minimize the risk of missing small lesions (21).

Currently, there is no established gold standard for the detection of intracranial extension or meningeal enhancement in cats and dogs with otitis media-interna. Fat-suppression techniques have proven helpful in identifying meningeal enhancement in dogs. However, no comparative study has been conducted between contrast-enhanced fat-suppressed-turbo spin echo-T1weighted (FS-TSE-T1W) and 3-dimensional-gradient recalled echo-T1weighted (3D-GRE-T1W) sequences in detecting meningeal enhancement.

The aims of this study were twofold. First, to compare the detection rate of meningeal enhancement in cats and dogs with otitis media-interna between two different T1 sequences, turbo spin echo T1W with fat-suppression (either spectral presaturation with inversion recovery (SPIR) or Dixon method) and 3D volumetric gradient-echo. Second, to assess inter- and intra-observer agreement for both techniques. The first hypothesis postulated that the detection rate for meningeal enhancement would be higher in FS-TSE-T1W compared to 3D-GRE-T1W sequences. The second hypothesis assumed a higher intra- and inter-observer agreement for detection of meningeal enhancement in FS-TSE-T1W than in 3D-GRE-T1W sequences.

## 2 Material and methods

### 2.1 Study population

A retrospective study was performed at the Clinic for Diagnostic Imaging, Vetsuisse Faculty of the University of Zurich. The first author (FG), a second-year resident of the European College of Veterinary Diagnostic Imaging, searched MRI studies in the local archive of patients with an imaging diagnosis of otitis (externa, media or interna), with or without intracranial extension. The following MRI-sequences had to be available: transverse T2W, T2W FLAIR, transverse T1W pre-contrast and FS-TSE-T1W (SPIR or Dixon) as well as 3D-GRE-T1W post-contrast sequences.

As no gold standard such as histopathological confirmation was available for classification of the cases and controls, a combination of the following features was used for classifying the control cases: presence of complete bilateral cochlear suppression on transverse T2-FLAIR together with the lack of neurological signs of vestibular disease (examined by a diplomate in veterinary neurology or resident under supervision) and normal CSF (if available). The transverse T2-FLAIR was only available for initial categorization by the author not related to image evaluation (KB), a diplomate of the European College of Veterinary Neurology. Additionally, medical records, signalment and results of CSF analysis (if available) were documented. MRI studies of the head of cats and dogs acquired between October 2017 and October 2023 were scrutinized. Follow-up studies were excluded from participation in this study. Additional intracranial pathologies, such as neoplasia, traumatic brain injury or encephalitis not related to the otitis represented equal exclusion criteria.

Patients were classified into two groups based on their original imaging diagnosis: (1) Otitis media/interna with or without meningeal enhancement, (2) Otitis media/externa without signs of otitis interna (control group). The author not related to the image evaluation (KB) anonymized and randomized the examinations.

### 2.2 Image acquisition

Magnetic resonance imaging was performed, with the patients under general anesthesia, with a 3T magnetic field scanner (Philips Ingenia, Philips AG, Zurich, Switzerland) using a 20-channel head/neck coil, or an eight-channel small extremity coil (Philips Ingenia, Philips AG, Zurich, Switzerland). Imaging parameters of the FS-TSE-T1W sequence included average repetition times (TRs) of 531–732 ms, dependent on the field of view (FOV), time to echo (TE) 8 ms, and slice thickness 2.75–3.0 mm. For the 3D-GRE-T1W sequence, TR was 11–13 ms, TE 5–6 ms, and slice thickness 0.7 mm.

Contrast medium was administered intravenously at 0.1 mmol/kg (2017–2019: 0.2 ml/kg Omniscan 0.5 mmol Gd-DTPA-BMA/ml, GE Healthcare AG, 8152 Glattbrugg, Switzerland. 2019–2023: 0.2 ml/kg Dotarem 0.5 mmol Gd/ml, Guerbet AG, 8050 Zürich, Switzerland).

### 2.3 Data analysis

The images were evaluated by five observers: four board certified veterinary radiologists (with 1–20 years of experience) and one second-year ECVDI resident. They were blinded to the signalment, original imaging diagnosis and clinical information, and reviewed the transverse T2W, pre-contrast 3D-GRE-T1W, post-contrast FS-TSE-T1W and 3D-GRE-T1W independently. A DICOM viewer software was used for this evaluation (HOROS, version 4.0.0., https://horosproject.org/). Evaluation of the studies took place twice by each individual observer at an interval of at least 4 weeks, with a separate examination of the FS-TSE-T1W and the 3D-GRE-T1W sequences, without side-by-side comparison. Evaluation criteria were the following: presence or absence of meningeal enhancement, degree (mild, moderate, severe) of meningeal enhancement, pachymeningeal enhancement, leptomeningeal enhancement or combined, presence or absence of meningeal splitting (18, 22), presence or absence of mass effect, degree of mass effect (mild, moderate, severe), distribution (focal, extension to contralateral side, generalized) and presence or absence of cochlear enhancement. An open annotation field for additional findings not contemplated in the evaluation list was available for the evaluators to annotate any relevant additional finding; the additional findings were then revised by the first and last author and descriptively included by consensus. Based on the results, assignment of the studies to one of two groups, either control group or otitis interna group, independent of meningeal enhancement, occurred.

### 2.4 Statistical analysis

Student's *T* test measured the detection rate of meningeal enhancement with a significance of *p* < 0.05. Fleiss' Kappa test measured the inter-observer agreement, including all analyzed images. To compare whether Fleiss' kappa values differed significantly between groups, a z-test was performed using the difference between kappa values divided by the standard error of the difference, assuming independent samples; *p*-values < 0.05 were considered statistically significant. Weighted kappa statistics were used to assess intra-observer agreement of meningeal enhancement between the 2-time assessment of each examiner. Data was normally distributed using the Kolmogorov-Smirnov test.

The data were prepared for statistical testing by the first author (FG). The statistical analysis was performed by the last author (AW) and the author not related to image evaluation (KB), both diplomates of the European Colleges of Veterinary Diagnostic Imaging and Veterinary Neurology, respectively, both holding a PhD degree with experience in biostatistics, using a dedicated software (Graphpad prism Version 10.4.2 and online extension https://www.graphpad.com/quickcalcs/kappa1/).

## 3 Results

### 3.1 Population

A total of 141 patients, including 61 dogs (23 females and 38 males) and 80 cats (40 females and males), met the inclusion criteria. Overall, 120 cases (46 dogs and 74 cats) were categorized as otitis media-interna and 21 (16 dogs and 5 cats) as control cases.

The canine breeds included French Bulldog (*n* = 24), Pug (*n* = 5), Cavalier King Charles Spaniel and mixed breed (4 each), Chihuahua, Boxer, Cocker Spaniel, Labrador, Welsh Springer Spaniel (2 each) and one of each of the following: American Staffordshire Terrier, Berger Blanc Suisse, Bernese Mountain Dog, St. Bernard, Border Collie, Continental Bulldog, Dalmatian, English Cocker Spaniel, Golden Retriever, Lagotto Romagnolo, Russian Toy Terrier, Staffordshire Bullterrier, Tibetan Terrier, West Highland White Terrier.

The age of dogs ranged from 1 to 16 years and weight from 2.3 to 83 kg.

The feline breeds included European Shorthair (*n* = 46), Maine Coon (*n* = 9), British Shorthair (*n* = 7), mixed breed and Persian (4 each), Bengal (*n* = 2), and one of each of the following: Burma, Exotic Shorthair, Norwegian Forest Cat, Russian Blue, Savannah, Scottish Fold Shorthair, Somali, Sphynx. The age of cats ranged from 4 months to 18 years and weight from 1.7 to 8.8 kg.

No histologic confirmation was available for any of the animals in our study population.

### 3.2 Detection rate of meningeal enhancement

On average, meningeal enhancement was detected in 34 cases in 3D-GRE-T1W and in 44 cases in FS-TSE-T1W of a total of 120 cases (118 otitis media-interna and two control cases). It is noted that meningeal enhancement was significantly more frequently detected in FS-TSE-T1W sequences compared to 3D-GRE-T1W by all observers with a *p*-value of 0.008 (first evaluation) and 0.003 (second evaluation). Table 1 provides an overview of the number of cases with detection of meningeal enhancement in the FS-TSE-T1W and 3D-GRE-T1W sequences according to observers in the first and second evaluation, respectively.

**Table 1:** Total number of cases rated positive for meningeal enhancement in the corresponding sequences, including cats and dogs in the first and second evaluation (1st and 2nd).

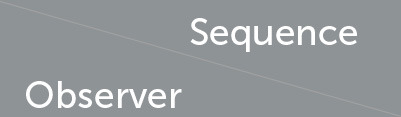	**3D-GRE-T1W**		**FS-TSE-T1W**	
	**1st evaluation**	**2nd evaluation**	**1st evaluation**	**2nd evaluation**
1	34	35	41	42
2	32	30	39	37
3	34	30	54	45
4	29	38	43	54
5	37	37	43	44

**Figure 1:**
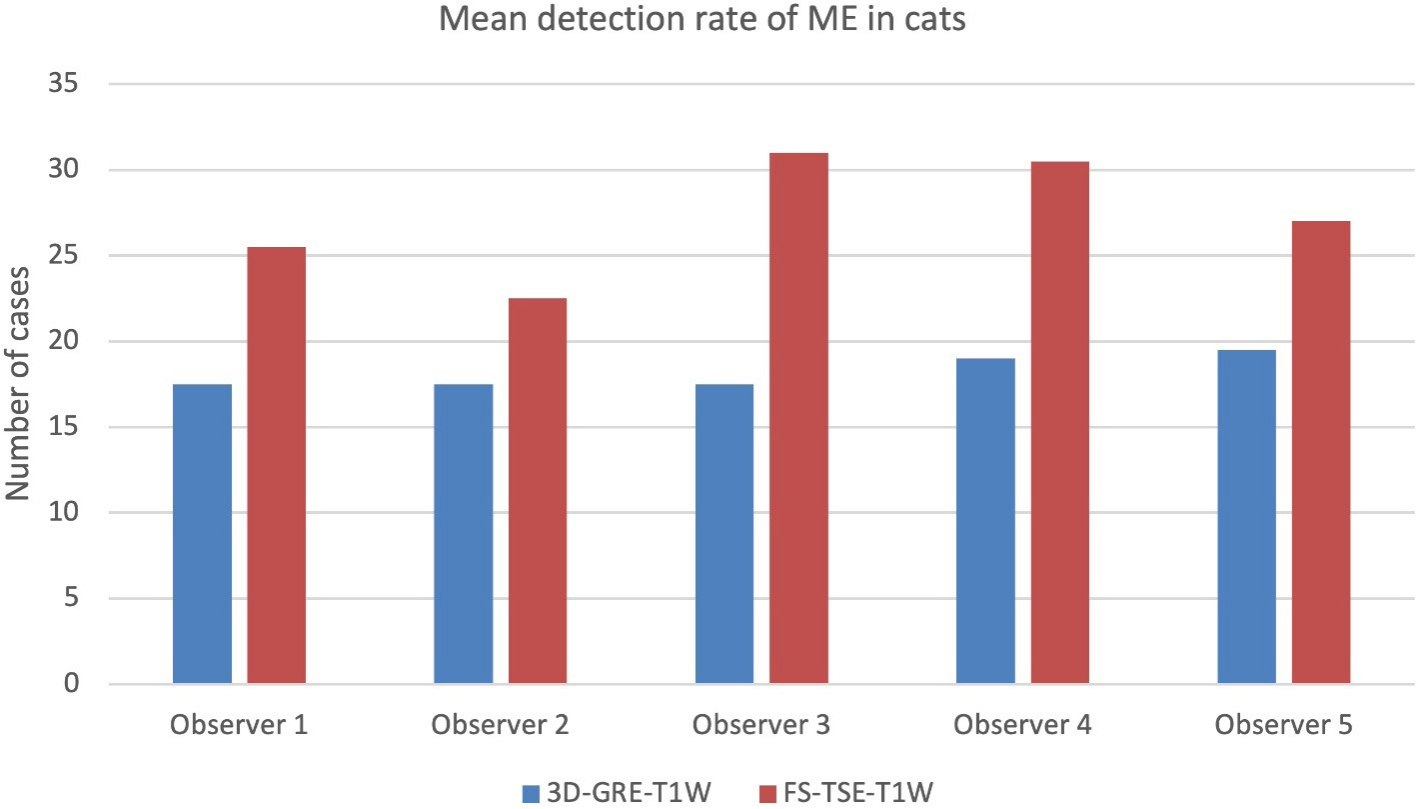
Mean detection rate (in number of cases) of meningeal enhancement in a total of 80 cats in 3D-GRE-T1W and FS-TSE-T1W sequences of all observers.

**Figure 2:**
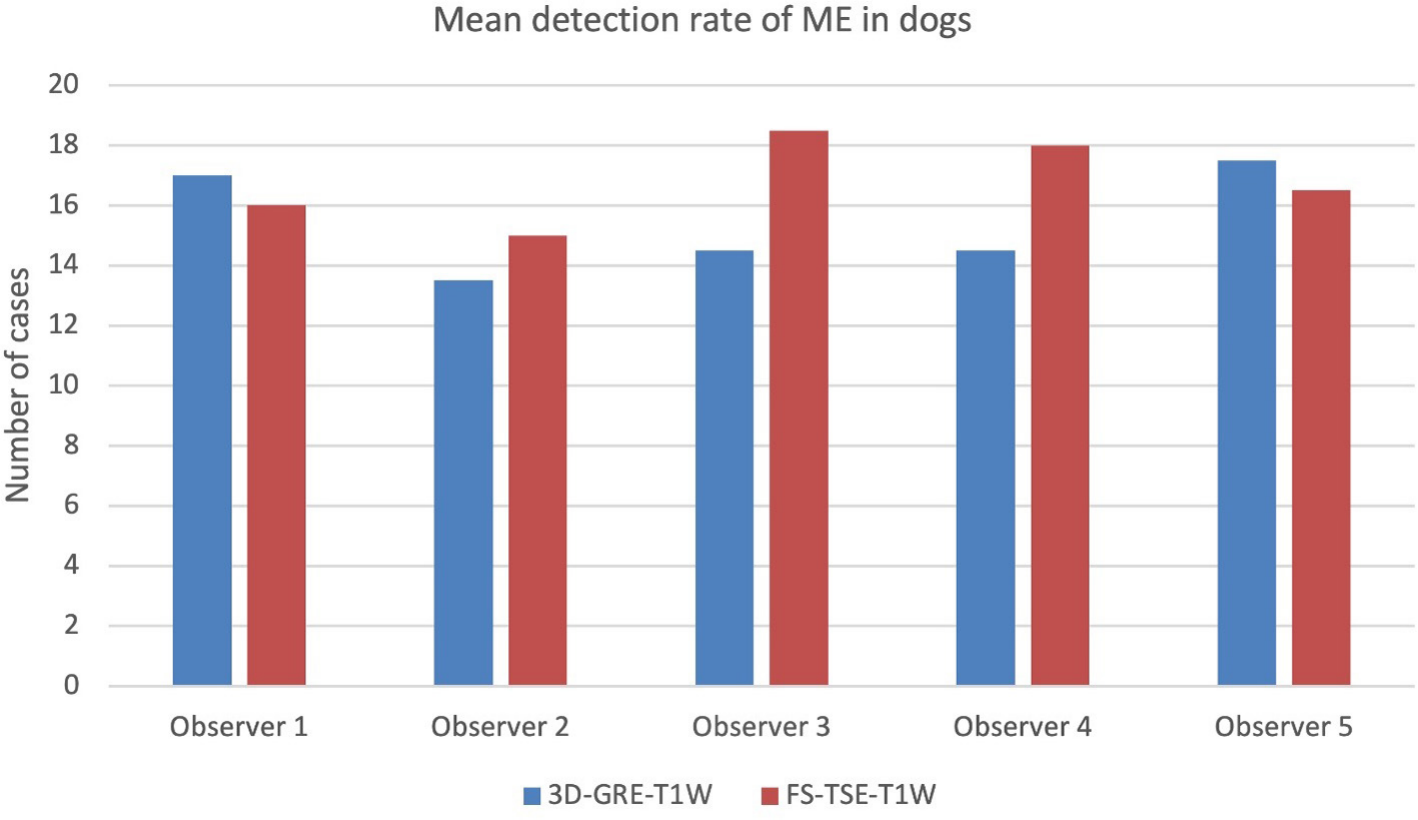
Mean detection rate (in number of cases) of meningeal enhancement in a total of 61 dogs in 3D-GRE-T1W and FS-TSE-T1W sequences of all observers.

When comparing detection rate of meningeal enhancement between cats and dogs, it was noted that detection rate of meningeal enhancement was significantly higher in cats in FS-TSE-T1W sequences compared to 3D-GRE-T1W sequences in the first and second evaluation (*p*-value of 0.001 and 0.004, respectively), but not in dogs (*p*-value of 0.175 and 0.122, respectively). Figures 1, 2 show the mean detection rate of meningeal enhancement in cats and dogs, respectively.

Cases were also subcategorized according to patient's weight, independent of the species. In patients with up to 5.5 kg (*n* = 70, 67 cats, 3 dogs) meningeal enhancement was detected in 48 cases, all being cats. In the category of 5.6–30.5 kg (*n* = 64, 13 cats; 51 dogs), meningeal enhancement was detected in 29 cases (4 cats; 25 dogs). In the last category with patients of 31–83 kg (*n* = 7 dogs), meningeal enhancement was detected in 5 cases.

### 3.3 Inter- and intra-observer agreement

Inter-observer agreement for the detection of meningeal enhancement was globally substantial for both, the FS-TSE-T1W (κ = 0.735) and the 3D-GRE-T1W (κ = 0.701) sequences (*p* = 0.089).

Regarding dogs, the inter-observer agreement was substantial for the FS-TSE-T1W (κ = 0.750) and the 3D-GRE-T1W sequence (κ = 0.724). For cats, the inter-observer agreement was substantial in both sequences and only marginally better in the FS-TSE-T1W (κ = 0.724) compared to the 3D-GRE-T1W (κ = 0.679) sequence. There were no significant differences when comparing kappa values relative to species (*p* = 0.99 for cats and *p* = 0.376 for dogs).

The intra-observer agreement between the first and second evaluation was substantial to near perfect for all observers in both sequences with kappa values given in Table 2.

**Table 2:** Intra-observer agreement of all observers in the corresponding sequence represented by kappa values.

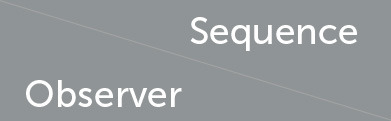	**3D-GRE-T1W**	**FS-TSE-T1W**
1	0.866	0.915
2	0.797	0.892
3	0.798	0.706
4	0.747	0.664
5	0.890	0.950

Complete or near complete agreement was reached in seven cases in the FS-TSE-T1W, exclusively.

**Figure 3:**
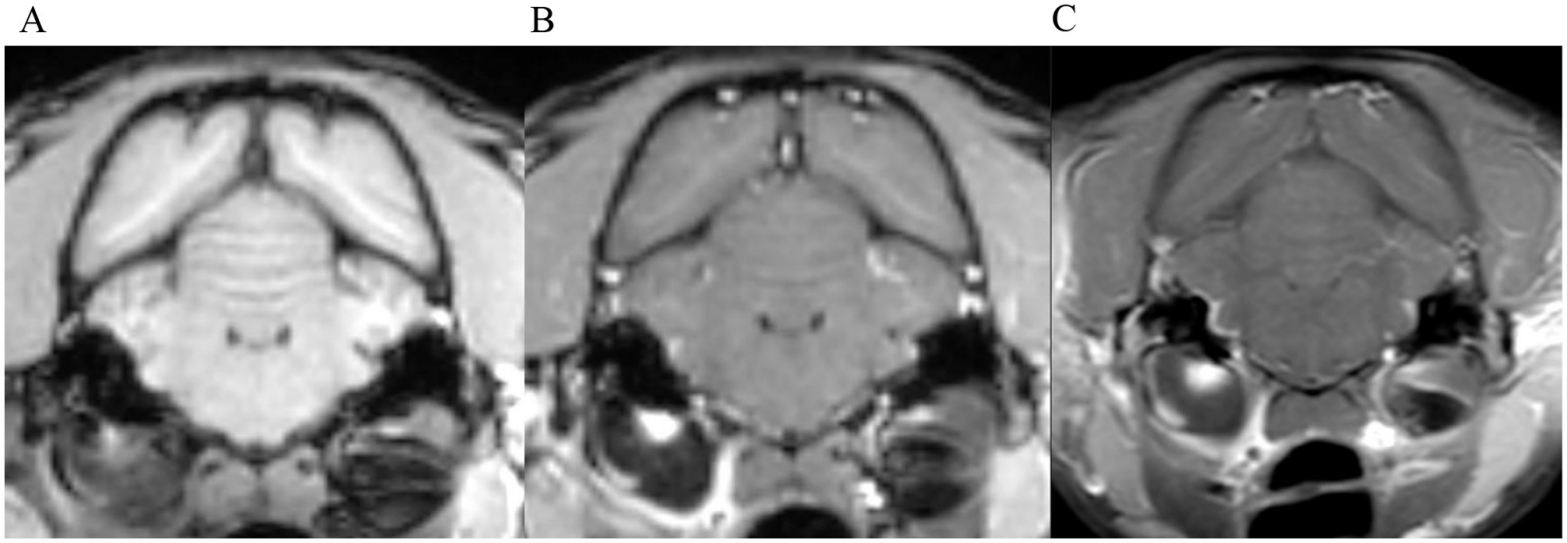
Transverse images at the level of the tympanic bullae. Example of perfect inter-observer agreement in the FS-TSE-T1W sequence in a cat. **(A)** is the T1W-pre-contrast image. 5/5 observers did not detect meningeal enhancement in the 3D-GRE-T1W **(B)**, but agreed on detection in the FS-TSE-T1W **(C)**. Meningeal enhancement was graded as focal and mild by all observers.

**Figure 4:**
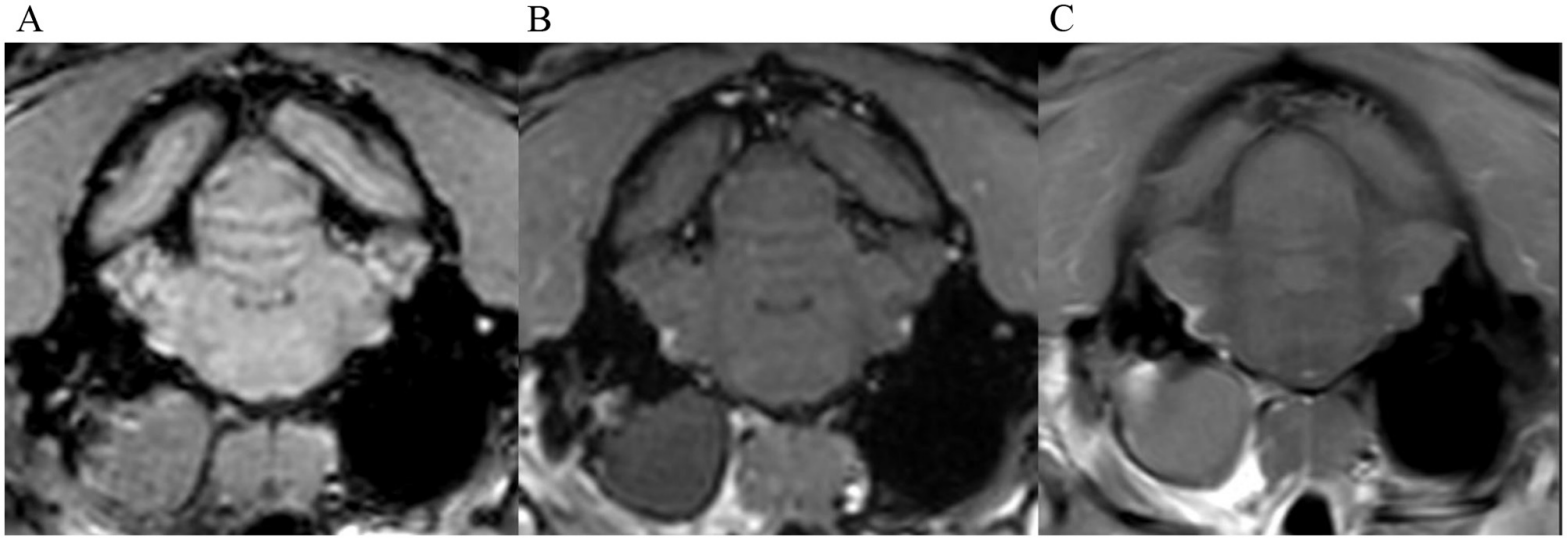
Transverse images at the level of the tympanic bullae. Example of perfect inter-observer agreement in the FS-TSE-T1W sequence in another cat. **(A)** is the T1W-pre-contrast image. 4/4 observers did not detect meningeal enhancement in the 3D-GRE-T1W **(B)**, but agreed on detection in the FS-TSE-T1W **(C)**. 3 observers graded it as focal and mild, while 1 observer graded it as focal and moderate enhancement.

**Figure 5:**
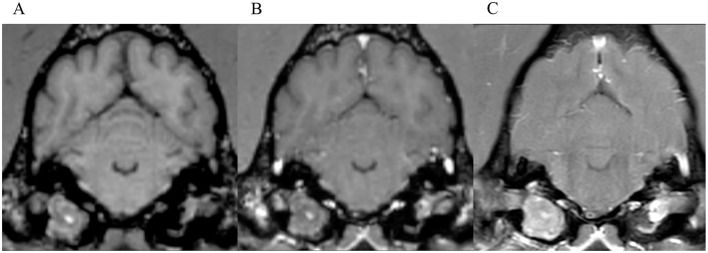
Transverse images at the level of the tympanic bullae. Example of a case where no inter-observer agreement was reached. **(A)** is the T1W-pre-contrast image. 1 of 5 observers did not interpret the 3D-GRE-T1W **(B)** as positive for meningeal enhancement but did interpret the FS-TSE-T1W sequence **(C)** as positive. No other observer detected meningeal enhancement in this case of a dog.

Examples of some of these cases can be found in Figures 3, 4. Meningeal enhancement was graded as focal and mild in both examples by 3 observers, while one observer graded the enhancement as focal and moderate (Figure 4). An example where no inter-observer agreement was reached can be found in Figure 5.

Meningeal enhancement was detected in two of the 21 control cases. In one of them by two observers, one in the 3D-GRE-T1W sequence and one in both sequences. In the other one, enhancement was detected only in the FS-TSE-T1W and only by one observer. In neither of these two control cases, the original imaging report stated meningeal enhancement.

### 3.4 Meningeal splitting

**Figure 6:**
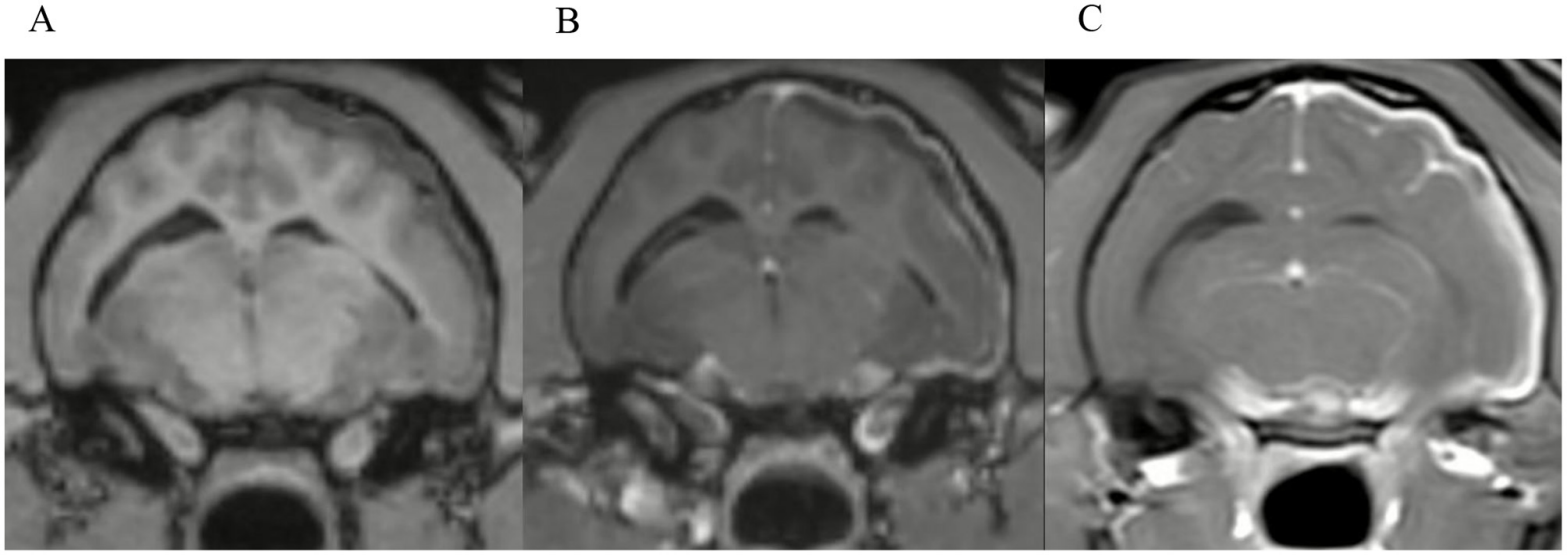
Transverse images at the level just caudal to the temporomandibular joints.Example of a case with marked meningeal enhancement and meningeal splitting in a cat. **(A)** is the T1W-pre-contrast image, **(B)** the 3D-GRE-T1W and **(C)** the FS-TSE-T1W.

**Figure 7:**
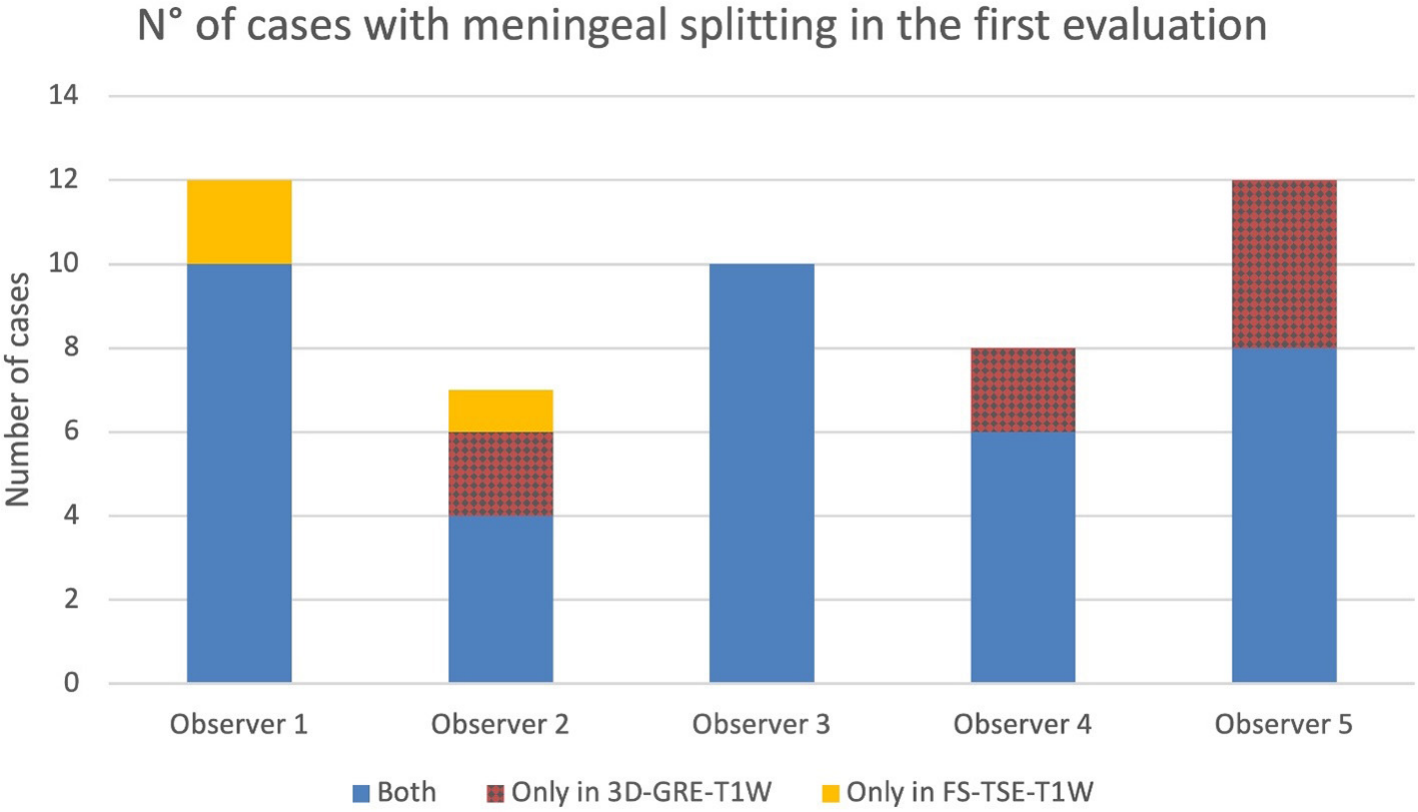
Number of cases in which meningeal splitting/empyema was detected by each observer in the corresponding sequences (Blue: both sequences, red: 3D-GRE-T1W and orange: FS-TSE-T1W) in the first evaluation.

**Figure 8:**
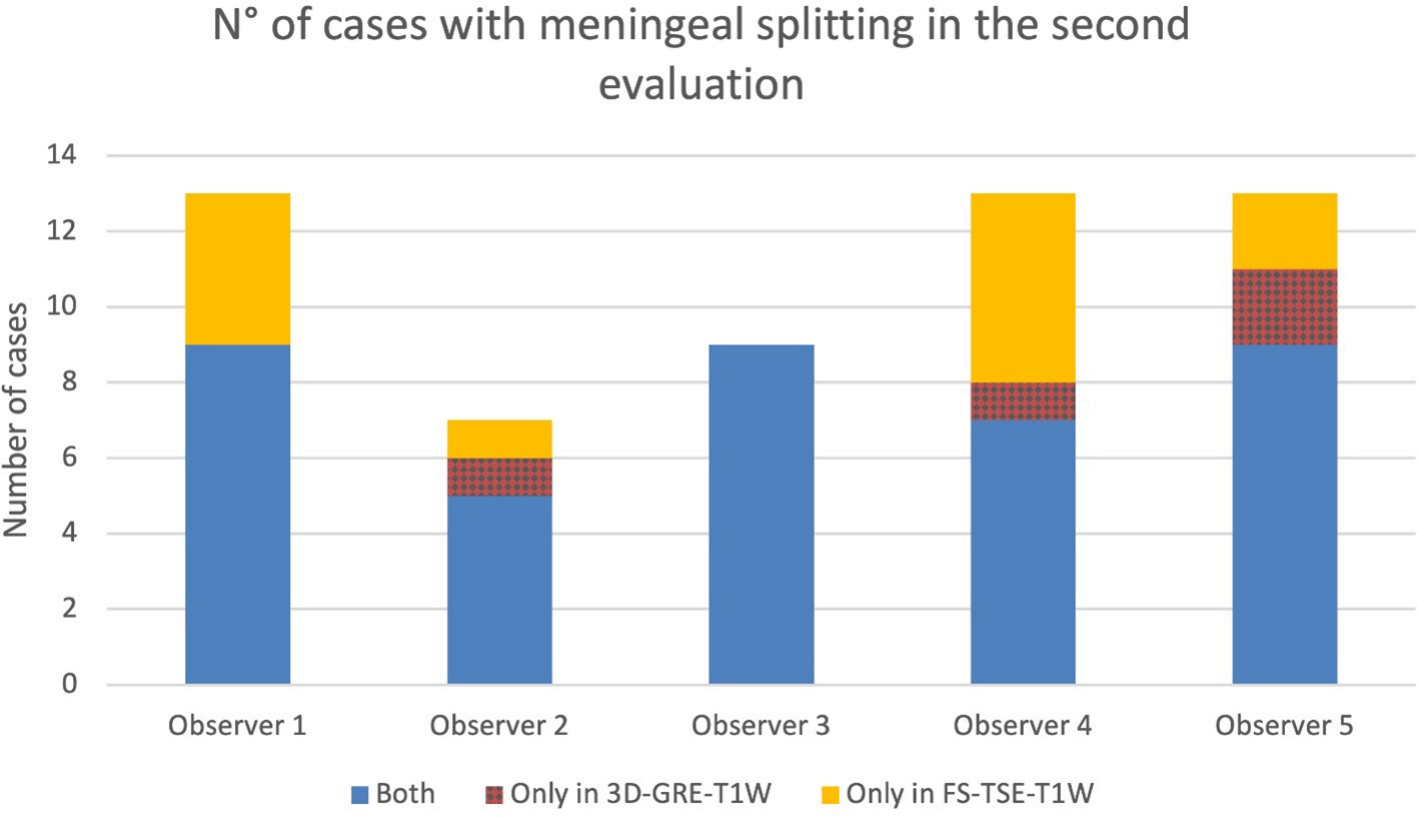
Number of cases in which meningeal splitting/empyema was detected by each observer in the corresponding sequences (Blue: both sequences, red: 3D-GRE-T1W and orange: FS-TSE-T1W) in the second evaluation.

In up to 13 cases observers detected meningeal splitting (Figures 6–8). This was seen either in both sequences, only in the 3D-GRE-T1W, or only in the FS-TSE-T1W sequence, as shown in Figures 7, 8. It is noted that if meningeal splitting was evident, it was seen most often in both sequences.

### 3.5 CSF analysis

Cerebrospinal fluid analysis was performed in 53 cases, being abnormal in 16 cases and unremarkable in 26 cases (Supplementary Table 1). In 11 cases, CSF analyses were excluded because of blood contamination.

### 3.6 Severity of meningeal enhancement

The grading of meningeal enhancement detected by observers 1–5 are displayed in Supplementary Table 2 by number of cases in the first and second evaluation. The table represents cases for both, the 3D-GRE-T1W and FS-TSE-T1W sequences.

**Figure 9:**
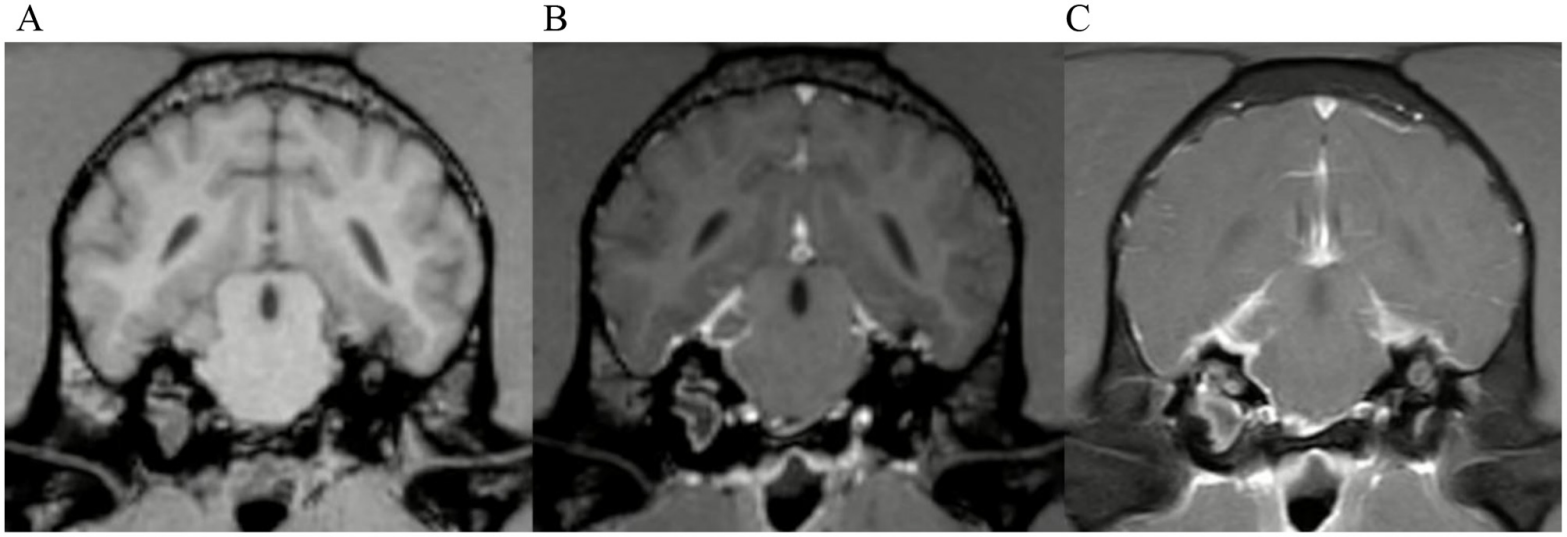
Transverse images at the level of the tympanic bullae. Example of a case with moderate meningeal enhancement in a dog, interpreted by observer 1. **(A)** is the T1W-pre-contrast image, **(B)** the 3D-GRE-T1W and **(C)** the FS-TSE-T1W.

Results on grading of mass effect, distribution and pattern of ME, cochlear enhancement and categorization of cases can be found in supplementary Tables 3, 4. Examples of moderate and marked meningeal enhancement can be seen in Figures 9, 6, respectively.

### 3.7 Additional observations

**Figure 10:**
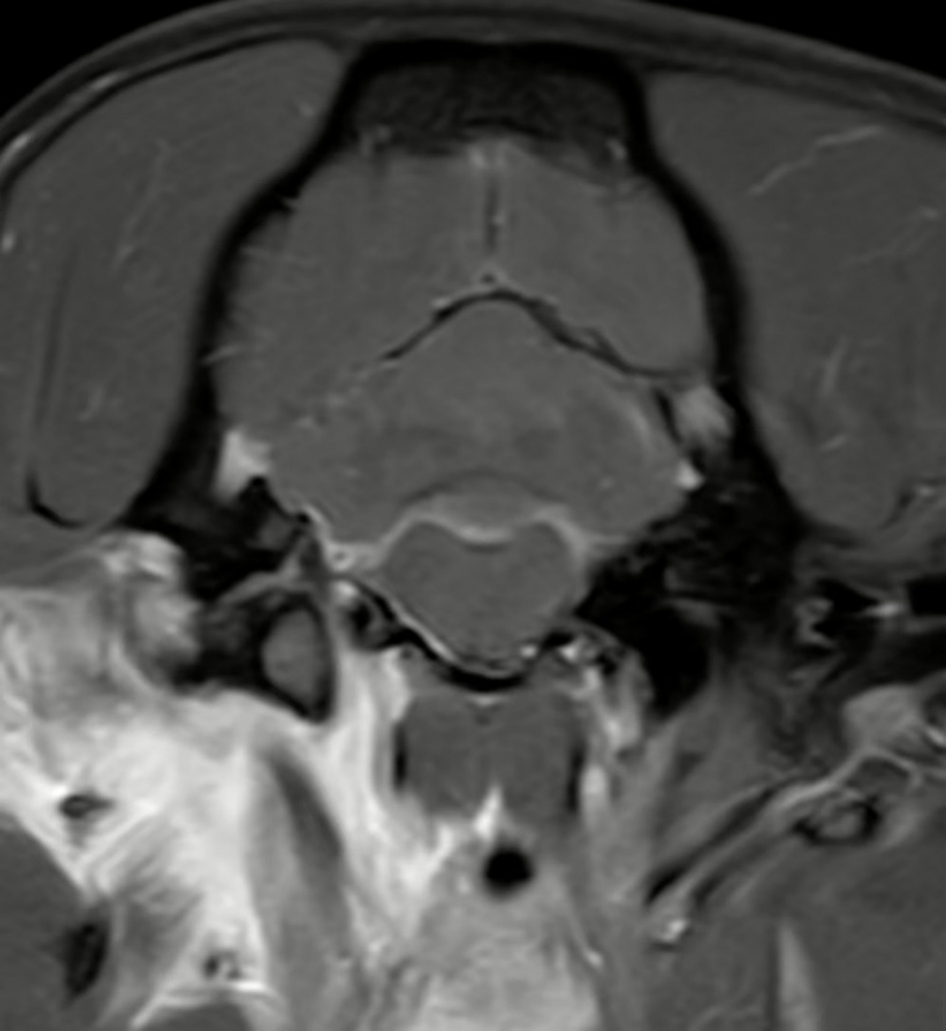
Transverse T1W-TSE post-contrast SPIR image at the level of the right jugular foramen. Example image of a dog with a presumed alternative route of intracranial extension through the right jugular foramen with regional meningeal enhancement. Note the marked contrast enhancement of the tissues around the right tympanic bulla (the right side of the image is the left side of the patient).

Two observers noted potential alternative routes of intracranial extension of disease besides the internal acoustic meatus. This was corroborated by the first and last authors in consensus. There was a total of 18 cases where inflammation appeared to extend through the jugular foramen (Figure 10). In 11 cases, the pathway of intracranial extension was suspected to be the oval foramen and in 5 cases both of the aforementioned routes were suspected to be involved. The identification of these presumed alternative routes was exclusively based on MRI findings such as the pattern of contrast enhancement, but no histopathological confirmation is available.

## 4 Discussion

This study aimed to compare the detection rate of meningeal enhancement in cases with otitis media-interna in cats and dogs between post-contrast FS-TSE-T1W and 3D-GRE-T1W sequences and to assess inter- and intra-observer agreement for both techniques. Overall, detection rate of meningeal enhancement was significantly higher in FS-TSE-T1W sequences compared to 3D-GRE-T1W sequences. No difference was found in inter- and intra-observer agreement and, grouped by species, the significance applied only to cats and not to dogs.

The higher detection rate prompted acceptance of the first hypothesis, being consistent with results of a previous study where chances to obtain a definitive diagnosis of meningeal enhancement were significantly higher with the use of post-contrast chemical fat-suppression compared to post-contrast transverse T1-weighted FLAIR sequences (2). Another study showed that hybrid fat-suppression technique (SPIR) have effectively highlighted meningeal enhancement in a dog with meningeal carcinomatosis (3).

There are several possible explanations for an increased detection rate with fat-suppression in our study, especially considering that detection rate was significantly higher in cats but not in dogs. First, the hyperintense appearance of fat within the calvarium on conventional spin echo sequences can mask nearby areas of contrast enhancement. Using fat-suppression techniques contrast enhancing tissues are highlighted due to effective suppression of adipose tissue (23). Fat-suppression also alters the image grayscale by eliminating the high signal from fat, allowing regions with intermediate to high signal intensity to occupy the upper end of the remaining dynamic range. This enhances contrast ratios and increases the visibility of gadolinium-enhancing lesions. When fat-suppression is used, the presence and extent of contrast enhancement should be interpreted cautiously, as the grayscale modification can exaggerate the apparent degree of enhancement (24). Second, volume averaging artefact due to thicker slices in FS-TSE-T1W sequences could have exacerbated the appearance of meningeal enhancement especially in smaller patient size like cats. The slice thickness in the FS-TSE-T1W sequences is larger compared to 3D-GRE-T1W sequences. In our study, slice thickness in FS-TSE-T1W sequences was around 2.5–2.8 mm with a spacing between slices of around 2.75–3.0 mm, while slice thickness and spacing between slices in the 3D-GRE-T1W sequences was in the submillimeter range. Another important factor to consider is that 3D-GRE-T1W sequences are particularly prone to artifacts caused by magnetic susceptibility differences such as air-bone-brain interfaces, as present at the level of tympanic bullae (25).

Given the lack of a histological confirmation of meningeal involvement, the increased detection rate must be interpreted with caution, as it may represent true meningeal pathology or the above-mentioned technical factors. However, the low number of false positives in the control group did not suggest over-reading, but true presence of meningeal pathology. A possible explanation that two of the control cases had a positive meningeal enhancement could rely on similar technical factors including volume averaging of enhancing meningeal vessels. Additionally, it has been stated that meningeal enhancement can be apparent in normal canine meninges (26, 27). In addition, diffuse contrast enhancement could also be caused by other diseases apart from otogenic intracranial infection (15, 18, 28).

While our study focused on the comparison between FS-TSE-T1W and 3D-GRE-T1W sequences, a similar, more recent study comparing fat-suppressed images with subtraction images found that subtraction offered no benefit over paired pre- and post-contrast fat-suppressed GRE images in the evaluation of the canine and feline brain (23). Meanwhile an earlier study found that subtraction images outperformed paired pre- and post-contrast T1-weighted spin echo images (29). False positive interpretation of pathological meningeal enhancement in subtraction images has been documented in the veterinary literature (23, 26).

Additionally, to avoid misregistration artefacts mimicking meningeal enhancement in a research setting, postprocessing coregistration of the images would have been needed to be applied, similarly as described in human medicine literature (30, 31).

These techniques fall outside the scope of the present study.

The present study shows that if CSF alterations are evident, MRI was able to detect meningeal enhancement in most of the cases and in both sequences. This could suggest a possible correlation of CSF alteration in patients with meningeal enhancement related to otitis media-interna. Nevertheless, different studies found no significant association between CSF results and meningeal enhancement (6, 14, 15). In one case with severe neutrophilic pleocytosis in the CSF, only one observer detected meningeal enhancement and only in the FS-TSE-T1W sequence while 4 observers did not detect meningeal enhancement at all. Therefore, relying solely on CSF analysis results or on MRI is not effective for detecting intracranial inflammatory extension.

In this study, a contrast medium dosage of 0.1 mmol/kg was used, following the institutional protocol. Increased sensitivity was found in dogs with experimentally induced meningitis when using higher doses of gadolinium (≥ 0.3 mmol/kg) (32). The higher detection rate of meningeal enhancement in the FS-TSE-T1W sequences in the present study might support the higher sensitivity of the FS-TSE-T1W compared to the 3D-GRE-T1W sequence. It remains open, if the use of fat-suppression can mitigate the increase in gadolinium dosage to detect meningeal disease.

The substantial to near perfect inter- and intra-observer agreement in both sequences for detection of meningeal enhancement resulted in the rejection of the second hypothesis. No significant differences have been found between inter-observer agreement on presence of meningeal enhancement in dogs and cats between both sequences.

It was initially assumed that a sequence with a lower detection rate would lead to reduced inter- and intra-observer agreement, as it offers less clear or consistent findings. We could not confirm this in our study. All observers, independent of the level of experience, detected consistently more enhancement in the FS-TSE-T1W sequence. This supports the idea that the FS-TSE-T1W could be more sensitive than the 3D-GRE-T1W in detecting mild meningeal enhancement. Additionally, enhancement in severe meningitis even leading to meningeal splitting was mostly evident in both sequences in this study.

Various human studies investigated the inter-observer agreement on meningeal enhancement.

One study compared contrast-enhanced 3D T1-SPACE (Sampling Perfection with Application optimized Contrast using different flip angle Evolution), a single slab three-dimensional TSE sequence, with 2D FLAIR and contrast-enhanced 2D T1-weighted sequences, finding that the 3D T1-SPACE showed the highest inter-observer agreement between raters and showed better detection rate of leptomeningeal metastasis (33).

One study comparing contrast-enhanced T2W-FLAIR and contrast-enhanced T1W sequences found that inter-observer agreement was almost perfect for both sequences in detection of meningitis. Only a slightly higher diagnostic confidence and inter-observer agreement was seen for qualitative assessment in the T2W-FLAIR sequence (34). Another study comparing contrast-enhanced T2-FLAIR with contrast-enhanced T1W with magnetization transfer and with fat-suppression found moderate inter-observer agreement for all three sequences (35). Finally, one study comparing post-contrast T2-FLAIR, 3D-T1-SPACE and T1W fat-suppressed images concluded that meningeal enhancement could be better appreciated in post-contrast FLAIR and can be added to routine MRI protocols in cases of suspected meningitis. The absence of contrast enhancement in vessels with slow blood flow represents the advantage of post-contrast T2-FLAIR. This allows clearer distinction between the meninges and nearby veins (36). Investigation of the potential use of these techniques in veterinary medicine for detection of meningeal enhancement could be considered for future prospective studies.

This study has several limitations, the major one being the lack of a histological confirmation of absence or presence of meningitis. In the absence of a gold standard, the evaluation was focused on demonstrating the ability of observers to agree on meningeal enhancement detection across sequences. The observed level of agreement supports validity of the chosen method. Furthermore, no confirmation is available on presumed alternative routes of intracranial extension of otitis media-interna. Furthermore, the MRI sequences were not standardized because of the retrospective nature of the study. The possibility of isolated instances of recall bias cannot completely be ruled out—such as when a reader might have previously encountered a patient during clinical work. This risk is considered to be minimal, because the readers were blinded to patient identity, clinical information, and other MRI sequences. Additionally, the images were randomized, and a time gap of several months to years existed between the patient's hospital visit and the image evaluation. Another limitation is the cognitive bias, since the observers were looking specifically for meningeal enhancement.

## 5 Conclusion

This study demonstrates a significantly increased detection rate of meningeal enhancement in fat-suppressed sequences in cats but not in dogs. Care should be taken to avoid over-interpreting the findings. Inter-observer agreement was substantial for both sequences, the FS-TSE-T1W and 3D-GRE-T1W while intra-observer agreement was substantial to near perfect for both sequences, without significant differences.

Based on the results of this investigation, adding a post-contrast FS-TSE-T1W sequence to the standard otitis protocol appears to be a valid option.

## Data Availability

The raw data supporting the conclusions of this article will be made available by the authors, without undue reservation.
