# Supplementary material for: Comparative study of meningeal enhancement in canine and feline otitis media-interna: 3D-gradient-echo vs. fat-suppressed turbo-spin-echo-T1-weighted sequences in MRI
Source: Front Vet Sci. 2025 Sep 30;12:1664006. doi: 10.3389/fvets.2025.1664006 (PMC12518113; doi:10.3389/fvets.2025.1664006)
Supplement: Supplementary file 1 [file Data_Sheet_1.pdf]

frontiers

Supplementary Material

1 Supplementary Data

1.1 Supplementary Figures

Meningeal enhancement / CSF result	Detected		Only 3D-GRE-T1W		Only FS-TSE-T1W		Both sequences	
	1st evaluation	2nd evaluation	1st evaluation	2nd evaluation	1st evaluation	2nd evaluation	1st evaluation	2nd evaluation
Mild lymphocytic pleocytosis	Yes	Yes	0	0	0	0	5	5
Moderate neutrophilic pleocytosis	Yes	Yes	0	0	0	0	5	5
	Yes	Yes	0	0	0	0	5	5
Severe neutrophilic pleocytosis	Yes	Yes	0	0	0	0	5	5
	Yes	Yes	0	0	1	1	0	1
Mild mixed pleocytosis	Yes	Yes	0	0	0	2	1	1
	Yes	Yes	0	0	2	1	0	1
Moderate lymphocytic pleocytosis	Yes	No	0	0	0	0	1	0
Mild albuminocytologic dissociation	Yes	Yes	0	0	1	1	0	0
Eosinophilic pleocytosis	Yes	Yes	0	0	0	0	5	5
Mild relative increase in neutrophiles	Yes	Yes	0	0	0	0	5	5
Mild neutrophilic pleocytosis	Yes	Yes	0	0	0	0	5	5
Mild mononuclear pleocytosis	Yes	Yes	0	0	2	0	3	5
High suspicion of cryptococcosis	No	Yes	0	0	0	1	0	0

Mild lymphocytic pleocytosis	No	No	0	0	0	0	0	0
	No	No	0	0	0	0	0	0
Unremarkable	Yes	No	0	0	1	0	0	0
	No	Yes	0	0	0	1	0	0
	Yes	Yes	1	0	2	1	0	0
	No	Yes	0	0	0	1	0	0
	Yes	Yes	0	0	3	4	1	0
	Yes	Yes	0	1	1	2	1	0
	Yes	No	0	0	1	0	0	0
	Yes	No	0	0	1	0	0	0
	Yes	No	1	0	0	0	0	0
	Yes	No	0	0	0	0	1	0
	No	Yes	0	1	0	0	0	0
15 unremarkable cases	No	No	0	0	0	0	0	0

Supplementary Table 1.
Cases with CSF analysis and corresponding result in addition to detection of meningeal enhancement by number of observers in the corresponding sequences. Of the 15 unremarkable cases without detection of meningeal enhancement, 5 belonged to the control group.

Grade \ Observer	1				2				3				4				5			
	1st		2nd		1st		2nd		1st		2nd		1st		2nd		1st		2nd	
	A	B	A	B	A	B	A	B	A	B	A	B	A	B	A	B	A	B	A	B
Mild	8	13	6	5	5	8	6	9	3	3	3	3	2	6	9	15	2	2	2	3
Moderate	1	5	4	4	4	2	2	2	6	6	6	6	7	3	3	5	5	4	4	2
Severe	7	7	3	3	4	4	4	4	0	0	0	0	0	3	7	6	2	2	3	4

Supplementary Table 2.
Number of cases with detected meningeal enhancement with respective grading (mild, moderate, severe) in the first and second evaluation in the 3D-GRE-T1W (A) and FS-TSE-T1W (B) sequences respectively.

Category \ Observer	1		2		3		4		5	
	3D-GRE-T1W	FS-FSE-T1W	3D-GRE-T1W	FS-FSE-T1W	3D-GRE-T1W	FS-FSE-T1W	3D-GRE-T1W	FS-FSE-T1W	3D-GRE-T1W	FS-FSE-T1W
Mild	7	9	5.5	8.5	3	3	5.5	10.5	2	2.5
Moderate	2.5	4.5	3	2	6	6	5	4	4.5	3
Severe	5	5	4	4	0	0	7	4.5	2.5	3
Focal	8.5	9	19	22.5	14	27.5	11	25	17.5	19
Contralateral	5	12	10.5	12.5	11	10	15.5	18.5	5.5	3
Diffuse	20.5	18	1.5	2	10	3	7	4.5	14.5	19.5

Supplementary Table 3.
Average number of cases with respective grading of mass effect (mild, moderate, severe) and distribution of meningeal enhancement (focal, contralateral, diffuse) between the first and second evaluation of all observers in the 3D-GRE-T1W and FS-FSE-T1W sequence.

Category \ Observer	1		2		3		4		5	
	3D-GRE-T1W	FS-FSE-T1W	3D-GRE-T1W	FS-FSE-T1W	3D-GRE-T1W	FS-FSE-T1W	3D-GRE-T1W	FS-FSE-T1W	3D-GRE-T1W	FS-FSE-T1W
PE	13	11	19.5	21	17	24.5	13	27	20	20
P-LE	21.5	30	11.5	17	14.5	23	18.5	19	10	13
Unclear	0	0	0.5	0	1	1.5	2	2.5	6.5	10.5
CE	101.5	100.5	58.5	N/A	104.5	109	32	58.5	86	102.5
N/CE	39.5	40.5	82	N/A	36.5	32	109	82.5	55	38.5
Control	38.5	39	81	N/A	36.5	31.5	61	46	45.5	36.5
Otitis interna	102.5	102	60	N/A	104.5	109.5	80	95	95.5	104.5

Supplementary Table 4.
Average number of cases with respective pattern of meningeal enhancement (PE = Pachymeningeal, P-LE = Pachy- and leptomeningeal, unclear), presence or absence of cochlear enhancement (CE = cochlear enhancement or N/CE = no cochlear enhancement) and categorization into control or otitis interna group in the 3D-GRE-T1W and FS-FSE-T1W sequences. Please note that not all results from observer 2 were available.
